# The cannabinoid receptor I (CB1) enhanced the osteogenic differentiation of BMSCs by rescue impaired mitochondrial metabolism function under inflammatory condition

**DOI:** 10.1186/s13287-022-02702-9

**Published:** 2022-01-21

**Authors:** Wanhao Yan, Le Li, Lihua Ge, Fengqiu Zhang, Zhipeng Fan, Lei Hu

**Affiliations:** 1 Laboratory of Molecular Signaling and Stem Cells Therapy, Beijing Key Laboratory of Tooth Regeneration and Function Reconstruction, Capital Medical University School of Stomatology, No. 4 Tiantanxili, Dongcheng District, Beijing, 100050 China; 2 Tsinghua University Hospital, Stomatological Disease Prevention and Control Center, Tsinghua University, Beijing, China; 3 Department of Periodontology, Capital Medical University School of Stomatology, Beijing, 100050 China; 4 Research Unit of Tooth Development and Regeneration, Chinese Academy of Medical Sciences, Beijing, China; 5 Salivary Gland Disease Center and Beijing Laboratory of Oral Health, Capital Medical University School of Stomatology, Beijing, 100050 China

**Keywords:** CB1, Bone marrow mesenchymal stem cells (BMSCs), Mitochondrial metabolism, Osteogenic differentiation, Inflammation

## Abstract

**Background:**

Periodontitis is a chronic infectious disease leading to bone resorption and periodontal tissue disruption under inflammatory stimulation. The osteogenic differentiation ability of mesenchymal stem cells (MSCs) is impaired under the inflammatory environment, which limits the effect of treatment. The cannabinoid receptor I (CB1) is the main effector of the endogenous cannabinoid system (ECS), and our previous study verified that CB1 could enhance the osteo/dentinogenic differentiation of dental MSCs, which might be a target for alveolar bone regeneration. However, the effect of CB1 on the osteogenic differentiation of MSCs derived from bone remains unknown. In present study, we investigated the role and mechanism of CB1 on mitochondrial function and osteogenic differentiation of human bone marrow mesenchymal stem cells (hBMSCs) under inflammatory environment.

**Methods:**

Alkaline phosphatase (ALP) activity, alizarin red staining, quantitative calcium analysis, and osteogenic markers were used to detect the osteogenic differentiation ability of BMSCs. Real-time RT-PCR and Western blot were used to detect the gene expression. Seahorse Cell Mito Stress Test was used to detect the oxygen consumption rate (OCR). JC-10 assay was used to determine the mitochondrial membrane potential (MMP).

**Results:**

CB1 increased osteogenic differentiation potential and mitochondrial energy metabolism, including the OCR, MMP, and enhanced the expressions of *Nrf1* and *Nrf2* in hBMSCs without or with TNF-α or INF-γ stimulation. Then, the inhibitor of mitochondrial electron transport chain (ETC), rotenone (ROT), inhibited the osteogenic differentiation in hBMSCs, and CB1 could rescue ROT impaired osteogenic differentiation potentials of hBMSCs without or with TNF-α or INF-γ stimulation. Activation of ETC by Coenzyme Q10 (CoQ10) could restore the impaired osteogenic differentiation of hBMSCs by depletion of CB1 without or with TNF-α or INF-γ stimulation. Mechanismly, CB1 could activate the JNK signaling pathway, p38 MAPK signaling pathway, and inhibit the Erk1/2 signaling pathway.

**Conclusions:**

The activating of CB1 enhanced the osteogenic differentiation by rescuing the mitochondrial metabolism function in hBMSCs under the inflammatory environment, suggesting that CB1 is a potential target for enhancing bone regeneration under the inflammatory environment.

**Supplementary Information:**

The online version contains supplementary material available at 10.1186/s13287-022-02702-9.

## Background

Periodontitis is a chronic infectious disease caused by bacteria and multiple factors, leading to alveolar bone resorption and periodontal tissue destruction under acute or chronic inflammatory stimulation [1]. The inflammatory microenvironment, with the character, upregulated tumor necrosis factor-alpha (TNF-α), interferon-gamma (INF-γ), is essential in the initiation, development, and healing of periodontitis [2, 3]. TNF-α contributes to osteoclastogenesis and alveolar bone resorption directly or indirectly through nuclear factor-κB (RANK) ligand. In addition, the stimulation of INF-γ on monocytes and lymphocytes overrides the direct inhibitory effect on osteoclasts, which leads to *P. gingivalis*-induced bone loss [4]. The current periodontitis treatment, depending on the number and function of residual mesenchymal stem cells (MSCs) in the periodontal region, cannot make the periodontal supporting tissue get ideal regeneration. Transplanting autologous or allogeneic MSCs into periodontitis sites can improve the regeneration of defective periodontal tissue [5–7], suggesting as the promising therapy for periodontitis. However, the inflammatory environment not only reduces the number of local MSCs in patients with periodontitis, but also impairs the autologous MSCs function, which makes it difficult to achieve the requirements of tissue regeneration [8]. Therefore, enhancing the function of MSCs under the inflammatory environment is critically important for the treatment of periodontitis.

It has shown that the endogenous cannabinoid system (ECS) plays a regulatory role in the healing of inflammatory tissue, and the cannabinoid receptor pathway is a significant target for regulating cannabinoid-driven periodontal immunology [9, 10]. The cannabinoid receptor I (CB1) is the main effector of the ECS, which is a member of the class A G protein-coupled receptor (GPCR) family, including a glycosylated extracellular amino-terminal (N-term) and an intracellular carboxyl-terminal (C-term) domain connected by seven transmembrane domains (7TM), three extracellular loops (ECL1, ECL2, and ECL3) and three intracellular loops (ICL1, ICL2, and ICL3), which involve multiple downstream signaling cascades to regulate different cellular activities and functions [11]. Besides, the study has reported that CB1 is located in the periodontal tissues, and its expression can be down-regulated by bacterial inflammation [9]. THC, the most effective cannabinoid with psychoactive effect, could activate CB1 to promote the migration of periodontal ligament fibroblast by enhancing the adhesion between cells and extracellular matrix [12]. Our previous study has found that CB1 enhanced the osteo/dentinogenic differentiation ability of MSCs derived from periodontal ligament tissue (PDLSCs) via the p38 MAPK and JNK pathway in the inflammatory environment [13]. The above studies suggest that CB1 is a potential target for periodontal tissue healing and alveolar bone regeneration. However, the mechanism of CB1 on the function of MSCs remains unclear.

CB1 is functionally located in the mitochondrial outer membrane (termed “mtCB1”) and regulates mitochondrial energy metabolism [11, 14]. After being activated by exogenous cannabinoids and in situ endogenous cannabinoids, mtCB1 can regulate the memory process via inducing intra-mitochondrial signal pathways involving G proteins, soluble adenylyl cyclase (sAC), and the protein kinase A (PKA), resulting in the decrease of mitochondrial complex I enzyme activity and respiration in neuronal mitochondria [15, 16]. Moreover, CB1 functionally exists in the mitochondria of striated and heart muscle and directly regulates intramitochondrial signal and respiration [11]. Mitochondria are important organelles for MSC energy metabolism and are the main places where carbohydrates, fat, and amino acids finally oxidize to produce ATP [17]. Recently, many studies have shown that the energy metabolism of mitochondria can directly regulate the function of MSCs through several mechanisms, including the redox reaction and energy metabolism process transformation in glycolysis and oxidative phosphorylation (OXPHOS), the change of mitochondrial membrane potential (MMP), mitochondrial biogenesis, and so on [18, 19]. Previous studies found the differentiation of MSCs is often accompanied by mitochondrial biogenesis, which is controlled by *PGC-1α*, then further activates the expression of *Nrf1*, *Nrf2*, and *mtTFA* to coordinate with DNA polymerase γ in order to promote mtDNA replication [20, 21]. Moreover, the study has reported that the differentiation of the MSCs is often accompanied by mitochondrial biogenesis and caused glycolysis to weaken and OXPHOS enhanced, in turn generating enough energy to meet the metabolic needs of the MSCs, indicating that mitochondrial biogenesis plays an important role in MSC mitochondrial function [22]. Meanwhile, the mitochondrial energy metabolism would be impaired under the inflammatory environment, which leads to the reduced function of MSCs. Study shows that gingival fibroblast cells from patients with periodontitis were impaired in OXPHOS and have a lower MMP [23]. Rats with periodontitis present severer mitochondrial dysfunction, including attended ATP production, decreased mitochondrial DNA (mtDNA) copy number, and reduced mitochondrial biology [24]. However, the effect and mechanism of CB1 on mitochondrial energy metabolism in MSCs which involved periodontal regeneration in the inflammatory environment are still unclear.

In this study, we used TNF-α and INF-γ to mimic an inflammatory environment as in our previous study [13], and we revealed the role and mechanism of CB1 in human bone marrow mesenchymal stem cells (hBMSCs) in the inflammatory environment. Our results found that CB1 enhanced the osteogenic differentiation of hBMSCs by rescue the mitochondrial energy metabolism function under the TNF-α and INF-γ stimulation.

## Methods

### Cell cultures

hBMSCs were purchased from Cyagen Biosciences (Guangzhou, China) and cultivated as our previously depicted [25]. In this study, 10 ng/ml TNF-α (R&D Systems, Minneapolis, USA) and 100 ng/ml INF-γ (R&D Systems) were used to mimic an inflammatory environment. 50 nM Rotenone (ROT; Sigma-Aldrich, St. Louis, MO, USA) and 30 μM Coenzyme Q10 (CoQ10; Sigma-Aldrich) were used to stimulate the hBMSCs. The selective CB1 antagonist, 10 μM AM251 (Cayman Chemical, Ann Arbor, MI, USA), p38 MAPK specific inhibitor, 20 μM SB203580 (MedChemExpress, Monmouth Junction, NJ, USA), JNK specific inhibitor, 20 μM SP600125 (Merck, Darmstadt, Germany) were used to stimulate hBMSCs.

### Plasmid construction and viral infection

The plasmid was constructed and the viral was infected as our previous study [25]. Human full-length CB1 cDNA was fused to a haemagglutinin (HA) tag and then inserted into the pQCXIN retroviral vector, and CB1 shRNA and control shRNA lentivirus were obtained as our previously depicted [13]. CB1 shRNA and control shRNA lentivirus were obtained from GenePharma. The targeted sequence for the control shRNA (Consh) was as follows: 5′-TTCTCCGAACGTGTCACGTTTC-3′, the targeted sequence for the CB1 shRNA 1 (CB1sh1) was as follows: 5-GCCGCAACGTGTTTCTGTTCA-3′, and the targeted sequence for the CB1 shRNA 2 (CB1sh2) was as follows: 5-GCAGACCAGGTGAACATTACA-3′.

### Reverse transcriptase-polymerase chain reaction (RT-PCR) and real-time RT-PCR

Total RNA extraction, cDNA synthesis, and real-time RT-PCR procedures were performed as previously described [25]. Real-time RT-PCR reactions were carried out according to the QuantiTect SYBR Green PCR kit (Qiagen, Hilden, Germany) using an Icycler iQ Multi-colour Real-time RT-PCR Detection System. The primers for specific genes are listed in Additional file 1: Table S1.

### Alkaline phosphatase (ALP) activity assay and Alizarin red detection

BMSCs were grown in the osteogenic-inducing medium as previously described [13]. The ALP activity was determined by the ALP activity kit (Sigma-Aldrich) according to the manufacturer’s protocol. To detect mineralization potential, hBMSCs were induced for 2 weeks, the fix of hBMSCs with 70% ethanol, the stain of hBMSCs with 2% alizarin red (Sigma-Aldrich), and the measurement of the final calcium level proceeded as previously described [13].

### Western blot analysis

The total protein extraction and the SDS–polyacrylamide gel electrophoresis tests proceeded as previously described [26]. The primary antibodies used in this study were anti-CB1 (Cat No. 93815; Cell Signalling Technology, Beverly, MA, USA), mouse monoclonal anti-HA (Clone No. C29F4; Cat No. MMS-101P; Covance, Princeton, NJ, USA), anti-phospho-p38 MAPK (Cat No. 4631; Cell Signalling Technology), anti-p38 MAPK (Cat No. 8690; Cell Signalling Technology), anti-phospho-JNK (Cat No. 4668; Cell Signalling Technology), anti-JNK (Cat No. 9258; Cell Signalling Technology), anti-phospho-Erk1/2 (Cat No. 4377S; Cell Signalling Technology) and anti-Erk1/2 (Cat No. 4695S; Cell Signalling Technology). The primary monoclonal antibodies for the housekeeping proteins were the monoclonal antibody against histone H3 (Cat No.10809; Santa Cruz Biotechnology) and β-actin (Cat No. C1313; Applygen, China).

### Oxygen consumption rate (OCR)

The OCR was assessed by Seahorse Cell Mito Stress Test in real-time using the 24 well Extracellular Flux Analyzer XF-24 (Agilent, Santa Clara, CA, USA) according to the manufacturer’s protocol. To stabilize the analyzer, Seahorse XFe24 Analyzer (Agilent) was turned on with wave software (Agilent) running overnight. Meanwhile, each well of the 24-well utility plate was filled with 1 ml calibration solution (Agilent) and immerse the sensor in the Seahorse XFe24 sensor cartridge (Agilent). And hBMSCs (8 × 10^4^/well) were seeded in the XF-24 plate before the experiment. On the day of the experiment, before starting measurements, hBMSCs were placed in a Seahorse XF DMEM medium and pre-incubated for 45 min in a non-CO2 incubator set to 37 °C. FluxPak injection ports were sequential inject the following compounds: oligomycin (final concentration 1.5 μM), fluoro-carbonyl cyanide phenylhydrazone (FCCP, final concentration 1.0 μM), and rotenone/antimycin A (each final concentration 1.0 μM), which were all provided as lyophilized powders in Mito Stress Test Kit (Agilent). Then hBMSCs were transferred to the XF-24 Extracellular Flux Analyzer. After three recordings, oligomycin, FCCP, and rotenone/antimycin A were injected into each well, three measurements of each OCR were recorded. The interval of all recordings was 8 min, and the running time of a standard mitochondrial stress test was about 100 min.

### Mitochondrial membrane potential (∆Ψm) assay

The MMP of hBMSCs was determined by the JC-10 assay (Solarbio, Beijing, China). JC-10 is a lipophilic cyanine cationic dye, which can selectively penetrate mitochondria and reversibly convert red fluorescence into green fluorescence when membrane potential decreases. Healthy hBMSCs have high membrane potential. When the MMP is high, JC-10 selectively aggregates in the mitochondrial matrix to form red fluorescence. While when the MMP is low, JC-10 is located as a monomer and showed green fluorescence. hBMSCs (2 × 10^4^/well) were seeded in 24-well plates and then incubated with JC-10 solution in the buffer in the dark for 20 min, then observed with the fluorescence microscope.

### Statistical analysis

SPSS 10 statistical software (SPSS Inc., Chicago, IL, USA) was used in all statistical calculations. The student’s *t* test or one-way ANOVA was performed to identify statistical significance, and *P* ≤ 0.05 was considered significant.

## Results

### CB1 promoted osteogenic differentiation of hBMSCs

**Fig. 1:**
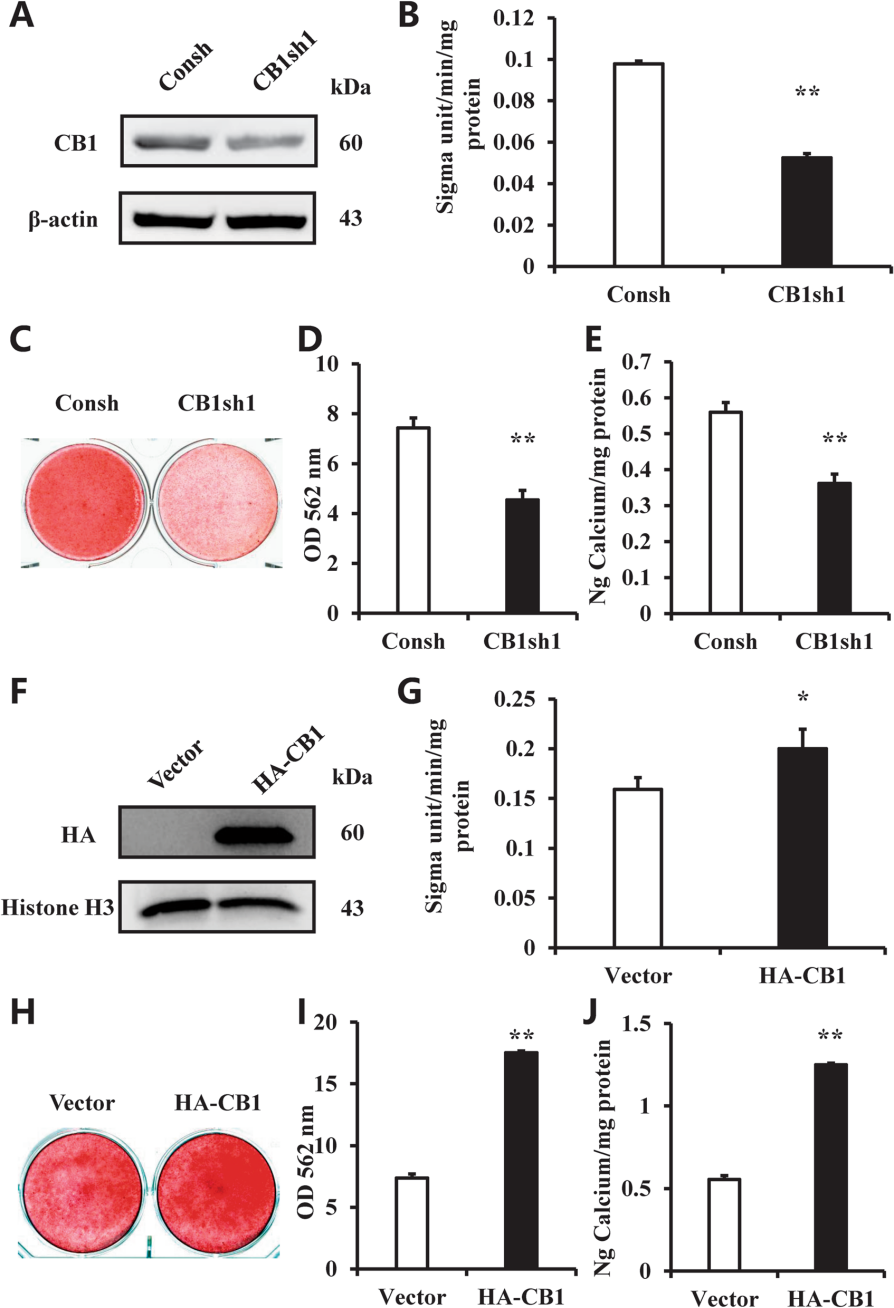
CB1 promoted the osteogenic differentiation of hBMSCs. **A** Western blot results showed the knockdown efficiency of CB1 shRNA1 in BMSCs. β-actin was used as an internal control. **B** The ALP activity assay in CB1 depleted hBMSCs. **C** Alizarin red staining in CB1 depleted hBMSCs. **D** OD values of the alizarin red staining in CB1 depleted hBMSCs. **E** Calcium quantitative analysis in CB1 depleted hBMSCs. **F** Western blot results showed the over-expression efficiency of HA-CB1 in BMSCs. β-actin was used as an internal control. **G** The ALP activity assay in CB1 over-expressing hBMSCs. **H** Alizarin red staining in CB1 over-expressing hBMSCs. **I** OD values of the alizarin red staining in CB1 over-expressing hBMSCs. **J** Calcium quantitative analysis in CB1 over-expressing hBMSCs. Student’s *t* test was performed to determine statistical significance. Error bars represent SD (*n* = 3). *P ≤ 0.05; **P ≤ 0.01

We used one of CB1 shRNA (CB1sh1) Lentivirus to delete the CB1 expression in hBMSCs. After using 1 μg/ml puromycin select for 3 days, western blot showed the CB1 was knockdown in hBMSCs (Fig. 1A). After being cultured with the osteogenic-inducing medium for 5 days, the ALP activity assay showed that the knockdown of CB1 by CB1sh1 decreased ALP activity of hBMSCs compared with the control group (Consh group) (Fig. 1B). Alizarin red staining, the calcium quantitative measurement also showed that CB1 deletion inhibited hBMSCs mineralization in vitro compared with the control group (Fig. 1C–E). Compared with the control group, the real-time RT-PCR results showed that *RUNX2* expression was significantly reduced at 2 weeks (Additional file 2: Fig. S1A), the *ALP* and *OSX* expressions were significantly decreased at 0, 1, and 2 weeks (Additional file 2: Fig. S1B, D), and the *OPN* expression was significantly reduced at 0 and 1 weeks after osteogenic induction in the CB1sh1 group compared with control group (Additional file 2: Fig. S1C). Furthermore, we used another CB1 shRNA (CB1sh2) to confirm CB1 function in hBMSCs. Western blot result showed that CB1 was knockdown in CB1sh2 group (Additional file 3: Fig. S2A). ALP activity, Alizarin red staining, the quantitative calcium measurement results confirmed that knockdown of CB1 by CB1sh2 inhibited the osteogenic differentiation potential of hBMSCs (Additional file 3: Fig. S2B–E).

Then, we inserted the HA-CB1 sequence into a retroviral vector, which was then transduced into hBMSCs by retroviral infection. After selection with 600 μg/ml G418 for 10 days, and western blot confirmed the overexpression efficiency of CB1 in hBMSCs (Fig. 1F). After 5 days of osteogenic induction, the ALP activity assay showed that CB1 overexpression enhanced ALP activity of hBMSCs compared with the control group (Vector group) (Fig. 1G). Alizarin red staining, calcium quantification revealed that the mineralization was significantly enhanced in BMSC-HA-CB1 cells in vitro of hBMSCs compared with the control group (Fig. 1H–J). The real-time RT-PCR results showed that *RUNX2* expression was significantly increased at 2 weeks (Additional file 4: Fig. S3A), the *ALP* expression was significantly increased at 0 week (Additional file 4: Fig. S3B), the *OPN* expression was significantly increased at 0, 1, and 2 weeks (Additional file 4: Fig. S3C), and the *OSX* expression were significantly increased at 0 and 1 weeks after osteogenic induction in CB1 overexpressed hBMSCs compared to the control group (Additional file 4: Fig. S3D).

### CB1 enhanced the OCR, MMP, and expressions of Nrf1 and Nrf2 in hBMSCs

**Fig. 2:**
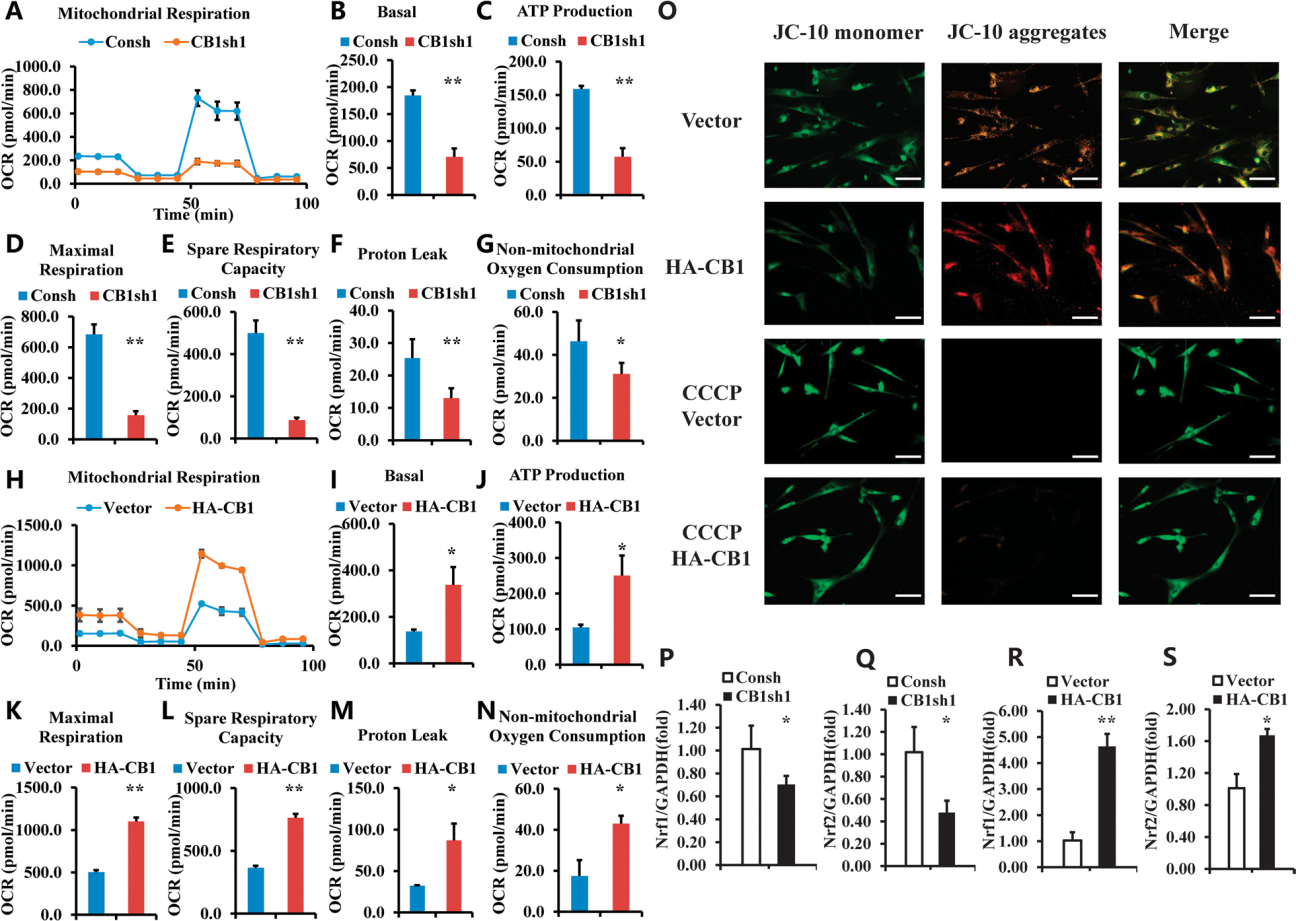
CB1 promoted the OCR, MMP and expressions of *Nrf1* and *Nrf2* in hBMSCs. **A**–**G** Knockdown of CB1 by CB1 shRNA1 inhibited the OCR in BMSCs. The Seahorse XFe24 analyzer showed the results of OCR curves (**A**), the basal OCR (**B**), ATP production (**C**), maximal respiration (**D**), spare respiratory capacity (**E**), proton leak (**F**) and non-mitochondrial oxygen consumption (**G**). **H**–**N** Role of over-expressing CB1 on the OCR in BMSCs. The Seahorse XFe24 analyzer showed the results of OCR curves (**H**), the basal OCR (**I**), ATP production (**J**), maximal respiration (**K**), spare respiratory capacity (**L**), proton leak (**M**) and non-mitochondrial oxygen consumption (**N**). **O** The JC-10 results in CB1 over-expressing BMSCs. CCCP was used as a positive control. Scale bar: 50 μm. **P–S** The Real-time RT-PCR results of *Nrf1* (**P**, **R**), and *Nrf2* (**Q**, **S**) in BMSCs. GAPDH was used as an internal control. Student’s *t* test was performed to determine statistical significance. Error bars represent SD (*n* = 3). *P ≤ 0.05; **P ≤ 0.01

Seahorse XFe24 analyzer was used to analyze the oxygen consumption of hBMSCs in a live cellular environment. The OCR curves are presented in Fig. 2A. Compared with the control group, it showed that the basal OCR, ATP production, maximal respiration, spare respiratory capacity, proton leak, and non-mitochondrial oxygen consumption were significantly reduced in the CB1sh1 group (Fig. 2B–G). Next, we used CB1 over-expressed hBMSCs to confirm CB1 function. We found that CB1 over-expressed hBMSCs showed significantly increased OCR compared to the control group (Fig. 2H). More specifically, the basal OCR, ATP production, maximal respiration, spare respiratory capacity, proton leak, and non-mitochondrial oxygen consumption were significantly increased in CB1 over-expressed hBMSCs compared to the control group (Fig. 2I–N).

Additionally, the JC-10 assay was used to analyze the mitochondrial membrane potential in CB1 over-expressed hBMSCs and the control group. CCCP is an OXPHOS uncoupler, which was used as a positive control to induce the decrease of MMP in BMSCs. As shown in Fig. 2O, CB1 over-expressed hBMSCs formed more JC-10 aggregates compared to the control group, indicated that the overexpression of CB1 promoted the MMP levels of hBMSCs. Next, we assessed the gene expression related to mitochondria metabolism and respiration. Real-time RT-PCR results showed that the knockdown of CB1 by CB1 shRNA1 significantly downregulated *Nrf1* and *Nrf2*, which regulate mitochondrial biogenesis compared to the control group (Fig. 2P, Q). Similarly, CB1 over-expressed hBMSCs increased the expression of *Nrf1* and *Nrf2* compared to the control group (Fig. 2R, S).

### CB1 restored the impaired osteogenic differentiation potential of hBMSCs by TNF-α or INF-γ

**Fig. 3:**
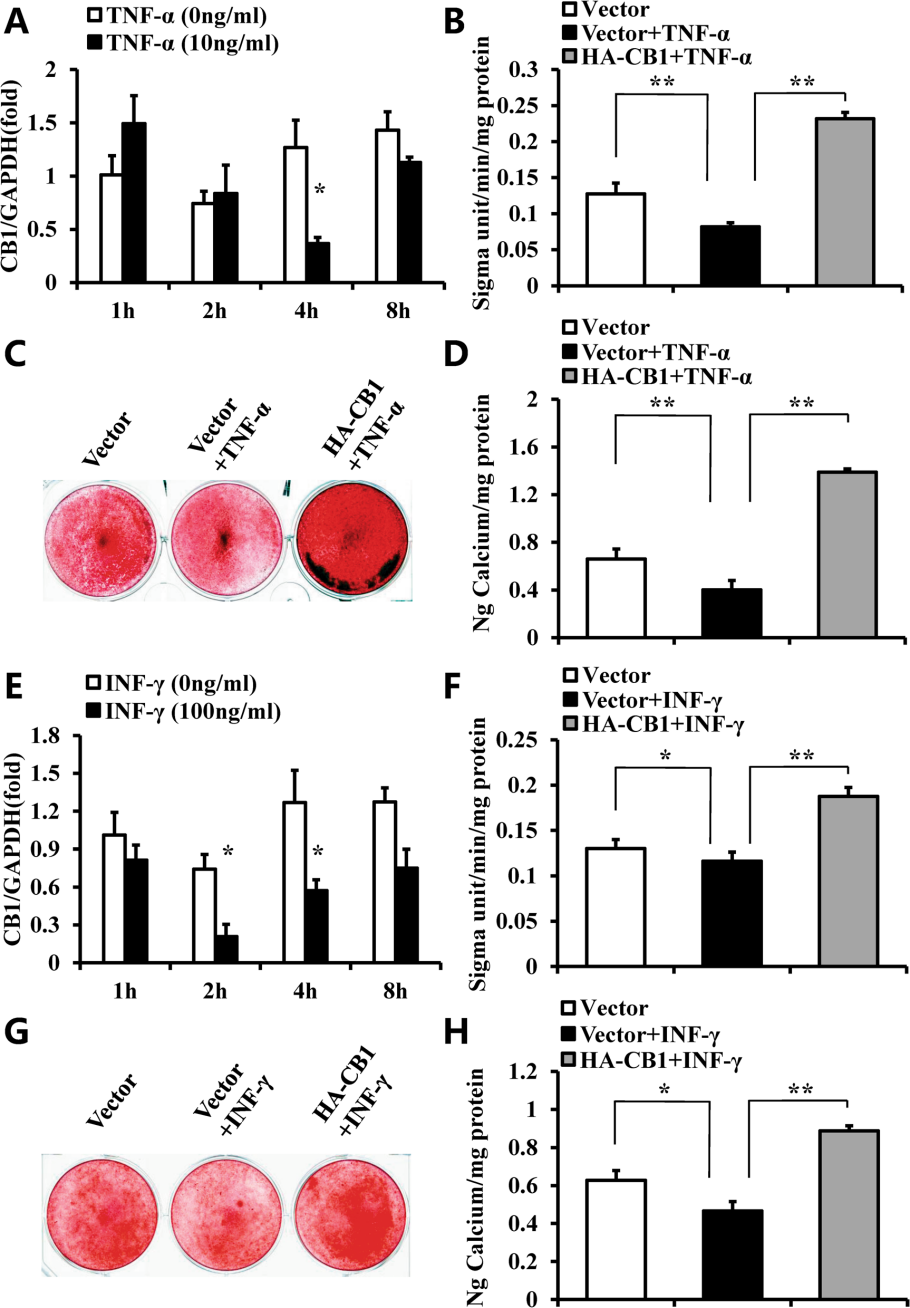
CB1 promoted the osteogenic differentiation of hBMSCs under TNF-α and INF-γ stimulation. **A**–**D** 10 ng/ml TNF-α was used to treat BMSCs. **A** Real-time RT-PCR results showed the expression of CB1 at 1, 2, 4 and 8 h after 10 ng/ml TNF-α treatment in BMSCs. **B** ALP activity assay. **C** Alizarin red staining. **D** Calcium quantitative analysis. **E–H** 100 ng/ml INF-γ was used to treat BMSCs. **E** Real-time RT-PCR results showed the expression of CB1 at 1, 2, 4 and 8 h after 100 ng/ml INF-γ treatment in BMSCs. **F** ALP activity assay. **G** Alizarin red staining. **H** Calcium quantitative analysis. GAPDH was used as an internal control. One-way ANOVA was performed to determine statistical significance. Error bars represent the SD (*n* = 3). *P ≤ 0.05; **P ≤ 0.01

The 10 ng/ml TNF-α or 100 ng/ml INF-γ were used to mimic an inflammatory environment as previously described [13]. Real-time RT-PCR results showed that the expression of CB1 in hBMSCs was reduced after 10 ng/ml TNF-α stimulation for 4 h compared with the untreated group (Fig. 3A). Then we investigate the role of CB1 in hBMSCs after TNF-α treatment. The results of ALP activity assay, alizarin red staining and the quantitative calcium analysis showed that 10 ng/ml TNF-α decreased the ALP activity and mineralization in hBMSCs, over-expression of CB1 could restore the impaired ALP activity and mineralization in hBMSCs by 10 ng/ml TNF-α treatment (Fig. 3B–D). Similarly, the expression of CB1 in hBMSCs was decreased at 2 h, and 4 h after 100 ng/ml INF-γ treatment (Fig. 3E). ALP activity, alizarin red staining, and the quantitative calcium measurement results showed that activation of CB1 could rescue the impaired ALP activity and mineralization of hBMSCs by 100 ng/ml INF-γ stimulation (Fig. 3F–H).

### CB1 increased the OCR, MMP, and expressions of Nrf1 and Nrf2 of hBMSCs which impaired after TNF-α or INF-γ stimulation

**Fig. 4:**
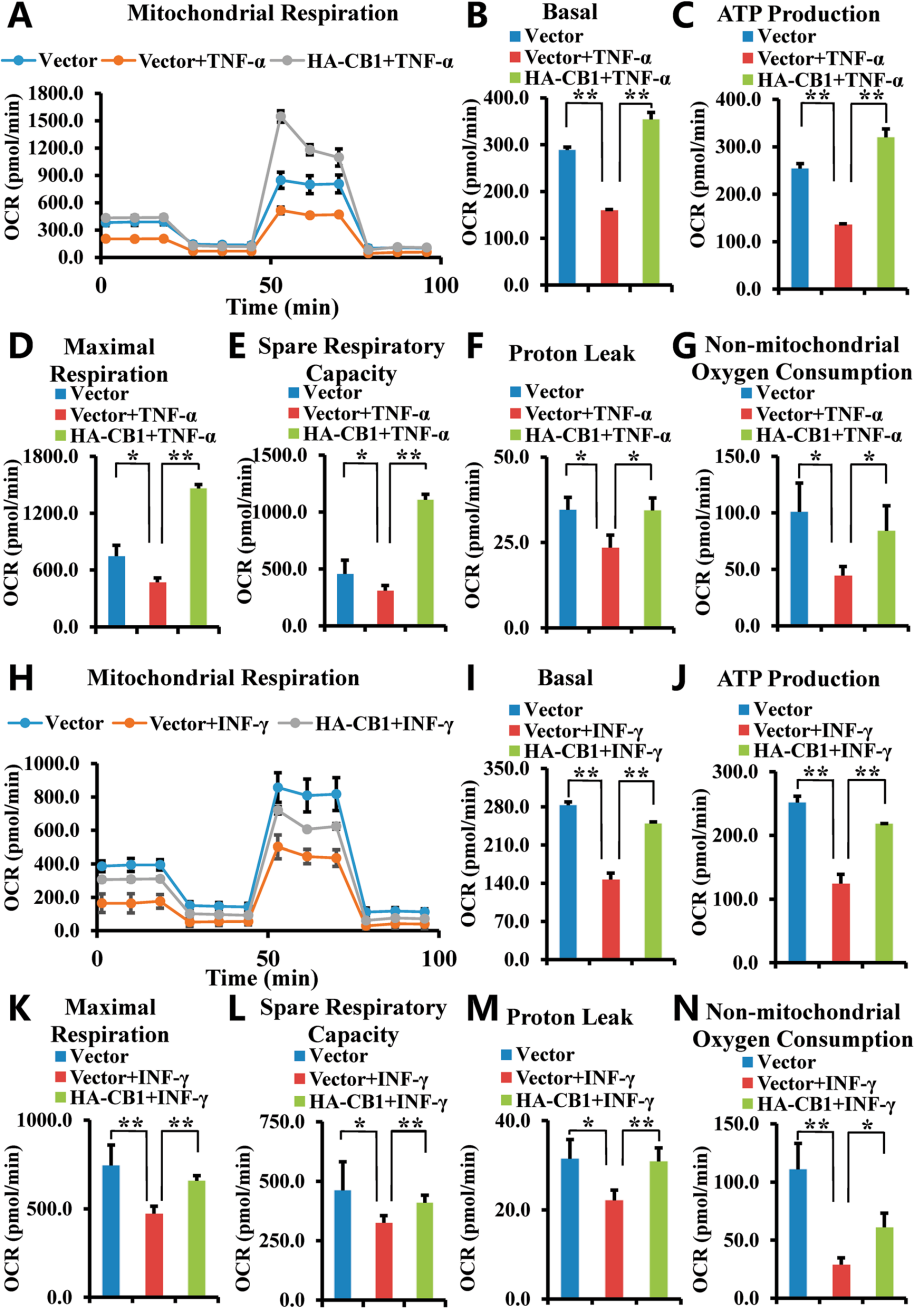
Over-expression of CB1 promoted the OCR in hBMSCs under TNF-α and INF-γ stimulation. **A**–**G** 10 ng/ml TNF-α was used to treat BMSCs for 24 h. The Seahorse XFe24 analyzer showed the results of OCR curves (**A**), the basal OCR (**B**), ATP production (**C**), maximal respiration (**D**), spare respiratory capacity (**E**), proton leak (**F**) and non-mitochondrial oxygen consumption (**G**). **H–N** 100 ng/ml INF-γ was used to treat BMSCs for 24 h. The Seahorse XFe24 analyzer showed the results of OCR curves (**H**), the basal OCR (**I**), ATP production (**J**), maximal respiration (**K**), spare respiratory capacity (**L**), proton leak (**M**) and non-mitochondrial oxygen consumption (**N**). The One-way ANOVA was performed to determine statistical significance. Error bars represent SD (*n* = 3). *P ≤ 0.05; **P ≤ 0.01

Next, we further verify CB1 function on OCR of hBMSCs under inflammatory conditions. Seahorse analysis results showed that 10 ng/ml TNF-α decreased the OCR in hBMSCs, over-expression of CB1 could restore this impaired OCR in hBMSCs induced by 10 ng/ml TNF-α treatment for 24 h (Fig. 4A), which include basal OCR, ATP production, maximal respiration, spare respiratory capacity, proton leak, and non-mitochondrial oxygen consumption (Fig. 4B–G). Similarly, the Seahorse analysis results showed that after 100 ng/ml INF-γ treatment for 24 h, INF-γ decreased the basal OCR, ATP production, maximal respiration, spare respiratory capacity, proton leak, and non-mitochondrial oxygen consumption in hBMSCs, and the overexpression of CB1 could rescue this impaired OCR (Fig. 4H–N).

**Fig. 5:**
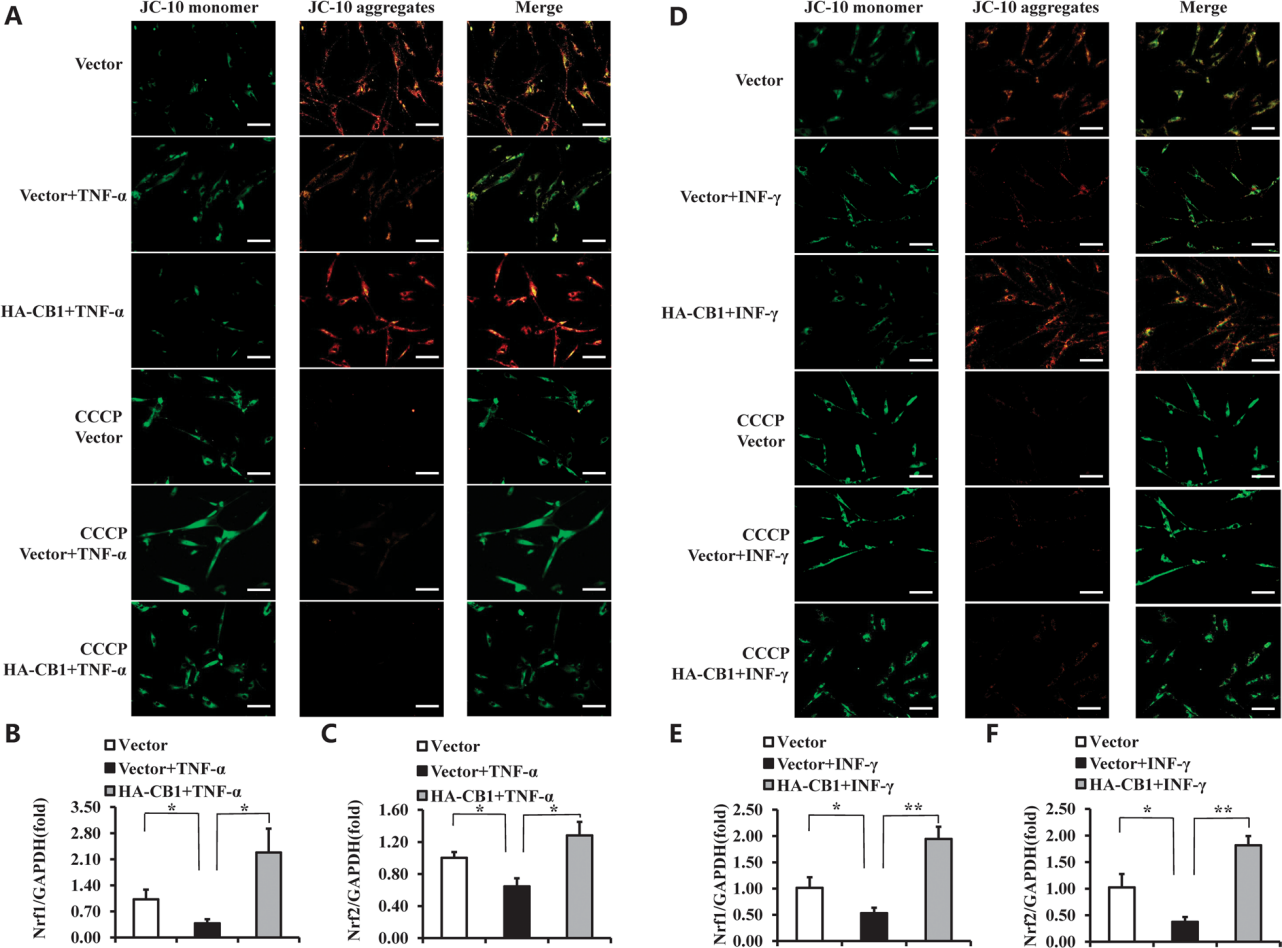
Over-expression of CB1 promoted the MMP and expressions of *Nrf1* and *Nrf2* in hBMSCs under TNF-α and INF-γ stimulation. **A** 10 ng/ml TNF-α was used to treat BMSCs for 24 h, the JC-10 results of BMSCs. CCCP was used as a positive control. Scale bar: 50 μm. **B**, **C** 10 ng/ml TNF-α was used to treat BMSCs for 4 h, the Real-time RT-PCR results of Nrf1 (**B**) and Nrf2 (**C**) expressions in BMSCs. **D** 100 ng/ml INF-γ was used to treat BMSCs for 24 h, the JC-10 results of BMSCs. CCCP was used as a positive control. Scale bar: 50 μm. **E**, **F** 10 ng/ml TNF-α was used to treat BMSCs for 4 h, the Real-time RT-PCR results of *Nrf1* (**E**) and *Nrf2* (**F**) expressions in BMSCs. GAPDH was used as an internal control. The One-way ANOVA was performed to determine statistical significance. Error bars represent SD (*n* = 3). *P ≤ 0.05; **P ≤ 0.01

Then, we further investigated CB1 function on MMP level and mitochondrial biogenesis ability of hBMSCs under inflammatory conditions. JC-10 assay indicated that the overexpression of CB1 promoted the form of JC-10 aggregates which was impaired under TNF-α for 24 h (Fig. 5A). Moreover, the real-time RT-PCR results showed that over-expression of CB1 significantly upregulated *Nrf1* and *Nrf2* in hBMSCs, which was impaired under TNF-α treatment for 4 h (Fig. 5B, C). Similarly, the MMP and the expressions of *Nrf1* and *Nrf2* were downregulated after 100 ng/ml INF-γ treatment for 4 h, and over-expression of CB1 could restore the MMP and the expressions of *Nrf1* and *Nrf2* (Fig. 5D–F).

### CB1 affected the osteogenic differentiation of hBMSCs through mitochondrial energy metabolism

**Fig. 6:**
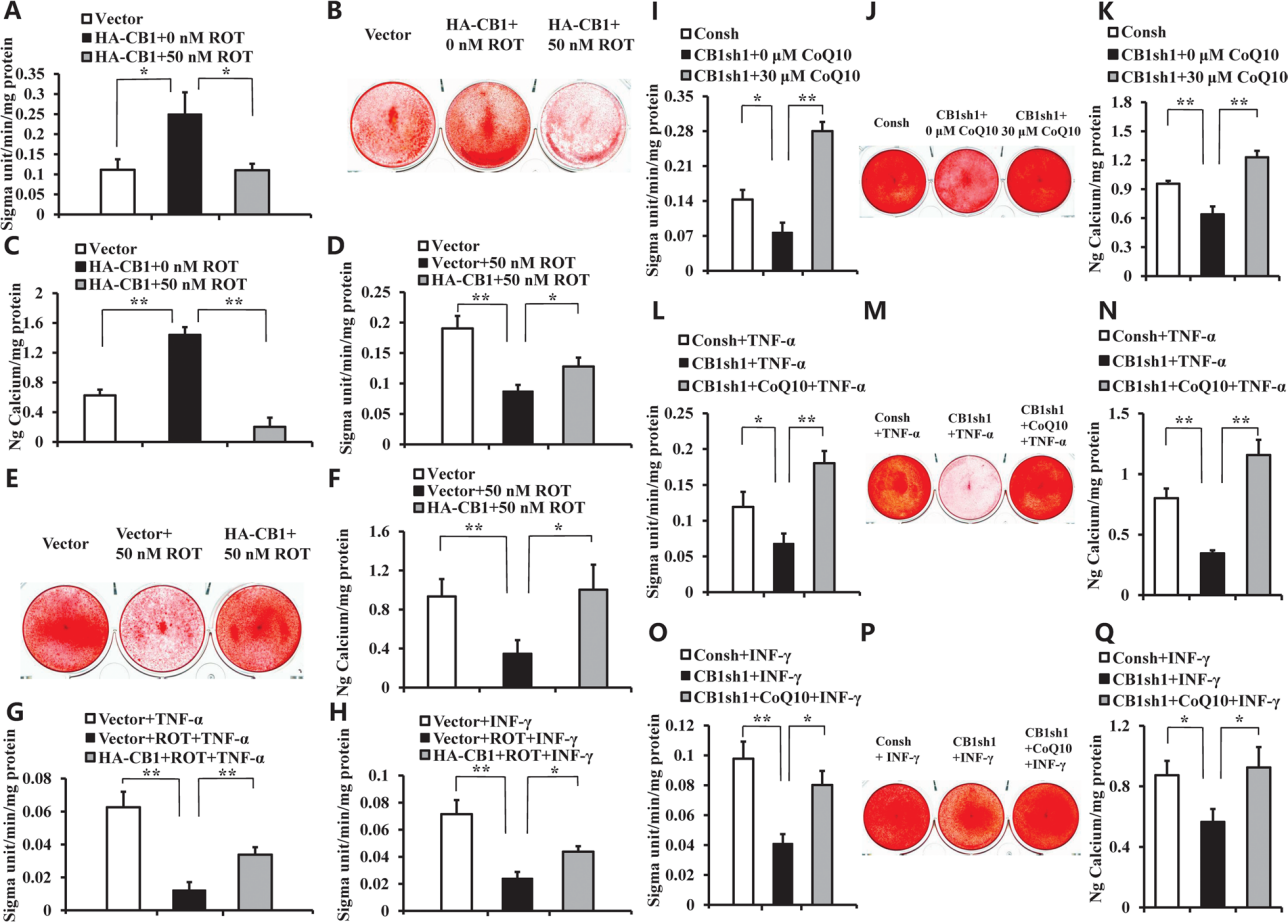
The function of ROT and CoQ10 on the osteogenic differentiation in hBMSCs. **A**–**F** 50 nM ROT was used to treat BMSCs. **A** The ALP activity results. **B** Alizarin red staining. **C** Calcium quantitative analysis. **D** The ALP activity results. **E** Alizarin red staining. **F** Calcium quantitative analysis. **G** 50 nM ROT and 10 ng/ml TNF-α were used to treat BMSCs. The ALP activity results. **H** 50 nM ROT and 100 ng/ml INF-γ were used to treat BMSCs. The ALP activity results. **I**–**K** 30 μM CoQ10 was used to treat CB1sh1 BMSCs. **I** The ALP activity results. **J** Alizarin red staining. **K** Calcium quantitative analysis. **L**–**N** 30 μM CoQ10 and 10 ng/ml TNF-α were used to treat CB1sh1 BMSCs. **L** The ALP activity results. **M** Alizarin red staining. **N** Calcium quantitative analysis. **O**–**Q** 30 μM CoQ10 and 100 ng/ml INF-γ were used to treat CB1sh1 BMSCs. **O** The ALP activity results. **P** Alizarin red staining. **Q** Calcium quantitative analysis. The One-way ANOVA was performed to determine statistical significance. Error bars represent SD (*n* = 3). *P ≤ 0.05; **P ≤ 0.01

We used an inhibitor of mitochondrial electron transport chain (ETC), ROT to clarify whether CB1 affects the osteogenic differentiation of hBMSCs through mitochondrial energy metabolism. After being cultured with osteogenic-inducing medium for 5 days, we discovered that 50 nM ROT reduced CB1-enhanced ALP activity of hBMSCs (Fig. 6A). Alizarin red staining and calcium quantitative assay showed that 50 nM ROT could inhibit ETC enhancement of mineralization in hBMSCs which induced by CB1 overexpression *in vitr*o (Fig. 6B, C). Moreover, the ALP activity, alizarin red staining, and quantitative calcium measurements revealed that 50 nM ROT impaired the osteogenic differentiaton potentials in hBMSCs, and over-expression of CB1 could restore the impaired ALP activity and mineralization by ROT treatment in hBMSCs (Fig. 6D–F). Then, we further investigated whether CB1 had the same role under the inflammatory environment. The results showed that 50 nM ROT inhibited the ALP activity, and CB1 could rescue the decreased ALP activity which caused by ROT in hBMSCs under TNF-α or INF-γ stimulation (Fig. 6G, H).

Then an essential component of the ETC, CoQ10 was used to investigate the function of CB1 in osteogenic differentiation of hBMSCs and the mitochondrial respiratory chain. After cultured hBMSCs with osteogenic-inducing medium for 5 days, we discovered that 30 μM CoQ10 could restore the decreased ALP activity which caused by knockdown of CB1 by CB1 shRNA1 in hBMSCs (Fig. 6I). Alizarin red staining and the calcium quantitative measurement also showed that CB1 deletion inhibited the mineralization of hBMSCs compared with the Control group, and the 30 μM CoQ10 promoted the mineralization in vitro in CB1 depleted hBMSCs (Fig. 6J, K). Similarly, we further detected the CB1 function under inflammatory conditions. The ALP activity assay, Alizarin red staining and the quantitative calcium measurement results indicated that 30 μM CoQ10 enhanced the osteogenic differentiation function of hBMSCs which was impaired by depletion of CB1 under TNF-α or INF-γ stimulation (Fig. 6L–Q).

### CB1 activated p38 MAPK and JNK signal pathways and repressed Erk1/2 signal pathway in BMSCs

**Fig. 7:**
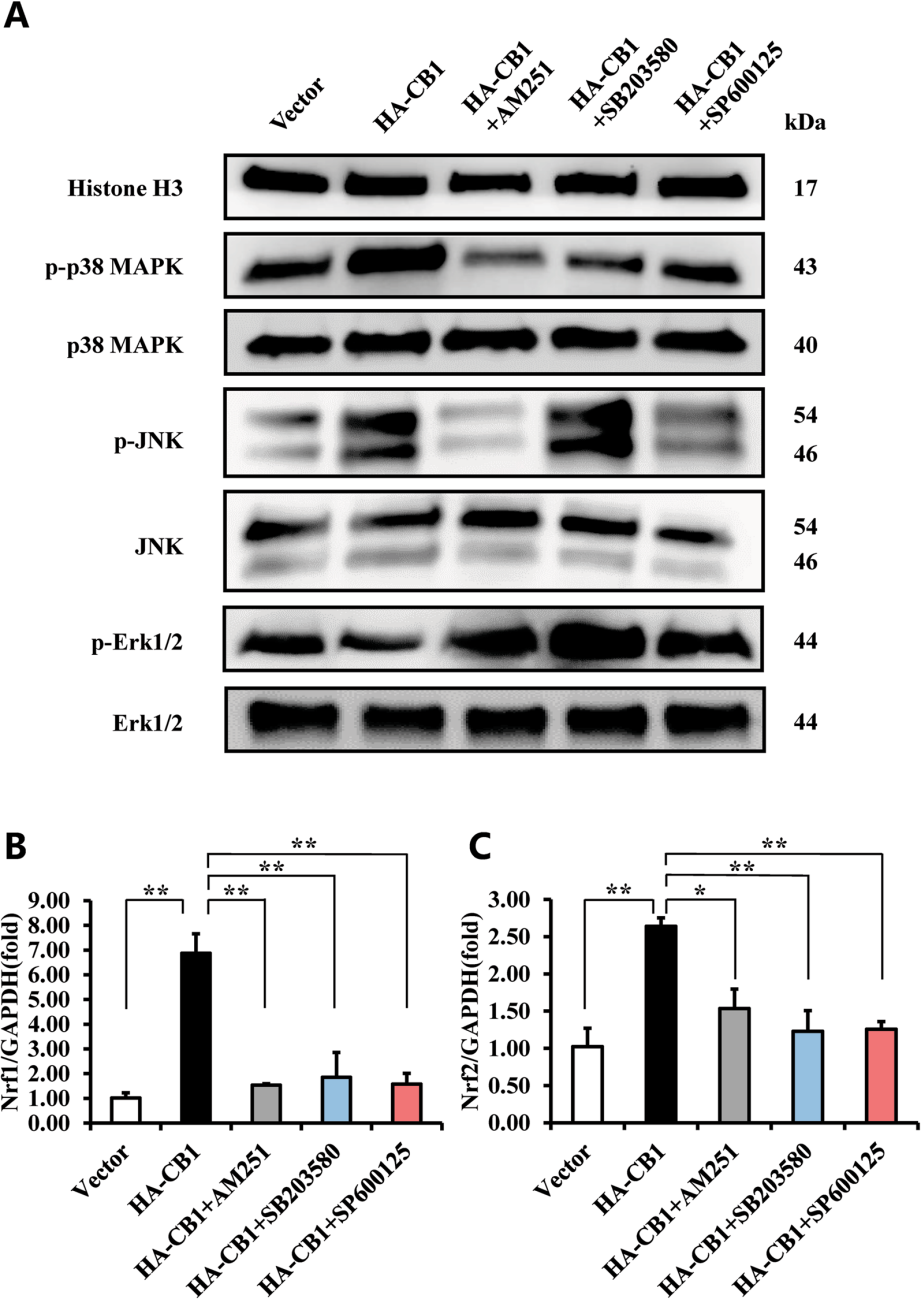
The effect of CB1 on MAPK signal pathways in hBMSCs. **A**–**C** 10 μM AM251 was used to treat the CB1 over-expressed BMSCs for 4 h, 20 μM SB203580 or 20 μM SP600125 was used to treat the CB1 over-expressed BMSCs for 2 h, Western blot results showed the expression of phosphorylated p38 MAPK, JNK and Erk1/2, and p38 MAPK, JNK, and Erk1/2 in BMSCs. Histone H3 was used as an internal control. **B**, **C** The Real-time RT-PCR results of *Nrf1* (**B**) and *Nrf2* (**C**) expressions in BMSCs. GAPDH was used as an internal control. One-way ANOVA was performed to determine statistical significance. Error bars represent the SD (*n* = 3). *P ≤ 0.05; **P ≤ 0.01

We further detected the MAPK signaling pathway in hBMSCs. Western blot results showed that overexpression of CB1 could enhance the phosphorylation of p38 MAPK and phosphorylated JNK, and decreased phosphorylated Erk1/2, while the total protein levels of p38 MAPK, JNK, and Erk1/2, p38 MAPK were not affected (Fig. 7A). Then, we blocked the CB1 with its specific inhibitor, AM251. Western blot results revealed 10 μM AM251 inhibited the increased phosphorylated p38 MAPK and phosphorylated JNK, and enhanced the inhibited phosphorylated Erk1/2 in CB1 overexpressing hBMSCs (Fig. 7A). Moreover, BMSCs were treated with 20 μM SB203580 (a specific inhibitor of p38 MAPK) for 2 h to block the p38 MAPK signaling pathway in the hBMSCs. Western blot results indicated that 20 μM SB203580 effectively suppressed the CB1-enhanced phosphorylated p38 MAPK, and increased the CB1-inhibited phosphorylated Erk1/2 in CB1 overexpressing hBMSCs (Fig. 7A). And then, BMSCs were treated with 20 μM SP600125 (a specific inhibitor of JNK) for 2 h to block the JNK signaling pathway in the hBMSCs. Interestingly, Western blot results showed that 20 μM SP600125 markedly suppressed the CB1-enhanced phosphorylated JNK and phosphorylated p38 MAPK, and increased the CB1-inhibited phosphorylated Erk1/2 (Fig. 7A).

Next, we detected whether CB1 activated the expressions of *Nrf1* and *Nrf2* in hBMSCs via p38 MAPK and JNK signal pathways. Real-time RT-PCR results showed that over-expression of CB1 enhanced the expressions of *Nrf1* and *Nrf2* in hBMSCs, which were significantly inhibited by 10 μM AM251, 20 μM SB203580, and 20 μM SP600125 in CB1 overexpressing hBMSCs (Fig. 7B, C).

## Discussion

Our previous study verified that CB1 could enhance the osteogenic differentiation of PDLSCs under inflammatory conditions [13]. However, normal autologous PDLSCs under inflammatory conditions decreased and their function is impaired, using autologous hBMSCs as seed cells has great advantages. Therefore, we further investigated the effect and mechanism of CB1 on the osteogenic differentiation of hBMSCs in the inflammatory environment. Firstly, the results of ALP activity, mineralization in vitro verified that knockdown of CB1 decreased osteogenic differentiation of hBMSCs, and over-expression of CB1 promoted osteogenic differentiation. It has been reported that CB1 is increased during the osteogenic differentiation of rat BMSCs, and is crucial for the survival of differentiated BMSCs during acute stress [26]. Moreover, aged CB1^−/−^ mice showed an increased age-related bone loss, partly due to impaired proliferation and differentiation of osteoblast [27, 28]. Study also reported that long-term treatment with topical Meth-AEA, a CB1 specific agonist, significantly diminished the alveolar bone loss and attenuated LPS-induced periodontitis in rats [29]. Our results are consistent with these findings, indicating CB1 promoted osteogenic differentiation in BMSCs.

Next, we explored the function of CB1 in hBMSCs in the inflammatory niche. TNF-α or INF-γ is the main inflammatory cytokine related to periodontitis. Previous study showed that TNF‐α at lower concentrations (0.01 and 0.1 ng/ml) positively enhanced expression levels of osteogenic transcription factors and bone marker genes of ST2 murine mesenchymal stem cells. Conversely, TNF‐α at concentrations above 1 ng/ml (10 and 100 ng/ml) dose-dependently displayed inhibitory effect on osteogenic differentiation [30]. In addition, it has been reported that a short-term treatment with TNF-α enhanced the stem cell phenotype, migration, and osteogenic differentiation ability of dental pulp stem cells (DPSCs) [31]. On the other hand, a long-term stimulation with TNF-α suppresses the differentiation ability of DPSCs [32], which indicates different concentrations and time of action of TNF-α has different effect on MSCs. It has been reported that PDLSCs from periodontitis patients (P-PDLSCs) showed impaired osteogenesis and regeneration ability, which could be mimicked in normal PDLSCs (N-PDLSCs) treated by 10 ng/ml TNF-α [33]. Moreover, in our previous study, we used 10 ng/ml TNF-α or 100 ng/ml INF-γ to treat N-PDLSCs and found 10 ng/ml TNF-α or 100 ng/ml INF-γ inhibited the osteogenic differentiation potentials [13], so we also chose these concentrations in present study. In accordance with the previous results in PDLSCs [13], our results also showed that 10 ng/ml TNF-α or 100 ng/ml INF-γ decreased the osteogenic differentiation ability of hBMSCs, while CB1 rescued the impaired osteogenic differentiation function in BMSCs caused by TNF-α or INF-γ. These results suggested that CB1 promoted osteogenic differentiation in BMSCs under inflammatory conditions.

Next, we explored the mechanism of CB1 on osteogenic differentiation of hBMSCs. Studies have reported that stem cells mainly rely on glycolysis for metabolism [34]. The differentiation of MSCs is accompanied by the decrease of mitochondrial glycolysis and the increase of OXPHOS, this bioenergy conversion plays an important role in the differentiation of MSCs [35]. The up-regulation of mitochondrial biogenesis and OXPHOS are the characteristics of MSC differentiation [20]. So we explored the effect of CB1 on mitochondrial respiration in hBMSCs. In present study, the OCR, MMP, and mitochondrial biogenesis of hBMSCs were analyzed. Our results showed that CB1 enhanced the OCR of hBMSCs, including basal OCR, ATP production, maximal respiration, spare respiratory capacity, proton leak, and non-mitochondrial oxygen consumption. Study has shown that Rimonabant, a CB1 antagonist in brain and peripheral, significantly increased mitochondrial biogenesis, and the OCR, consisting of basal OCR, maximal respiration, proton leak, and ATP production was enhanced in 3T3-L1 adipocytes [36]. These indicated that CB1 has different effects in different cell types. MMP is a good indicator of mitochondrial activity as it reflects the process of electron transport and OXPHOS and ATP production [37]. Therefore, we investigated the role of CB1 on MMP in hBMSCs. Our result has found that overexpression of CB1 promoted the MMP levels in hBMSCs, which is consistent with the results of ATP production in hBMSCs. It has been reported that stimulated CB1 by ACEA, AEA, and 2-AG downregulated mitochondrial biogenesis, including the expression of *PGC-1α*, *Nrf1*, and *mtTFA* in cultured mouse white adipocytes [38]. However, our results showed that *Nrf1* and *Nrf2* expressions were decreased in CB1 depleted hBMSCs, and increased in CB1 over-expressed hBMSCs. Nrf1 was an initial transcription factor identified for the induction of mitochondrial biogenesis by PGC-1α, a dominant negative allele of Nrf1 blocked the effects of PGC-1α on mitochondrial biogenesis [39]. Moreover, Nrf1 and Nrf2 are important contributors to the increase in transcription of key mitochondrial enzymes, and they have been shown to interact with *mtTFA*, which drives transcription and replication of mtDNA, suggesting that Nrf1 and Nrf2 play a key role in mitochondrial biogenesis [40]. Taken above, these up-regulating *Nrf1* and *Nrf2* by CB1 activation indicated that CB1 might enhance mitochondrial biogenesis of hBMSCs.

It has been reported that the metabolic conversion from glycolysis to OXPHOS is inhibited under inflammatory stimulation, and TNF-α repairs the osteogenic differentiation and OXPHOS of hBMSCs [41]. We further analyzed the OCR, MMP, and mitochondrial biogenesis of hBMSCs under inflammatory conditions. We found that 10 ng/ml TNF-α or 100 ng/ml INF-γ inhibited the OCR of hBMSCs, including basal OCR, ATP production, maximal respiration, spare respiratory capacity, proton leak, and non-mitochondrial oxygen consumption. Moreover, 10 ng/ml TNF-α or 100 ng/ml INF-γ decreased the MMP level, and *Nrf1* and *Nrf2* expressions of hBMSCs. Furthermore, over-expression of CB1 could rescue the inhibition of OCR, the MMP level, and *Nrf1* and *Nrf2* expressions caused by TNF-α or INF-γ stimulation. This suggests that activation of CB1 might increase the mitochondrial biogenesis and OXPHOS of hBMSCs in the inflammatory niche.

Study has shown that during the osteogenesis induction of the hBMSCs, the OCR and ATP production significantly increase [42, 43]. Inhibition of the metabolic switch via respiratory inhibitors can cause impaired osteogenesis differentiation [44]. Then, we explored whether CB1 enhanced the osteogenic differentiation of hBMSCs by affecting mitochondrial energy metabolism. In our study, ROT was used to block the ETC, the results of ALP activity and mineralization verified that inhibition of ETC decreased osteogenic differentiation of hBMSCs. Because of the cytotoxicity of ROT, the continuous inhibition of cell respiration leads to apoptosis, which leads to the failure of osteogenic differentiation of BMSCs for two weeks under inflammatory conditions. Therefore, we only analyzed the ALP activity of hBMSCs under inflammatory conditions, and the results showed that block ETC by ROT decreased osteogenic differentiation of hBMSCs, and the over-expression of CB1 promoted osteogenic differentiation of hBMSCs under inflammatory conditions. It has been reported that LPS-treated fibroblasts induced mitochondrial dysfunction, decreased OCR, ATP levels, and mitochondrial biogenesis, while the addition of mitochondrial ETC activator, CoQ10 generated a significant restoration by improving mitochondrial biogenesis in vitro, indicating that the energy metabolism of mitochondria is impaired in periodontitis [45]. Then, CoQ10 was used to further confirm the function of CB1. Our results showed that CoQ10 could restore the impaired osteogenic differentiation of hBMSCs by depletion of CB1 without or with TNF-α or INF-γ stimulation. The above results indicated that CB1 affected the osteogenic differentiation of hBMSCs by affecting mitochondrial energy metabolism under inflammatory conditions.

In addition, our previous study has shown that CB1 activated the p38 MAPK and JNK signals while inhibiting Erk1/2 signal in PDLSCs [13]. Therefore, we detected these signaling pathways in hBMSCs. Similarly, we found that CB1 could active p38 MAPK and JNK signaling pathway, while inhibiting the Erk1/2 signaling pathway in hBMSCs. And addition of CB1 specific inhibitor, AM251, further verified these results. Interestingly, treatment with SP600125, a specific inhibitor of JNK, the CB1-activated p38 MAPK signaling pathway seemed to be inhibited, and the CB1-inhibited Erk1/2 signaling pathway seemed to be activated in CB1 over-expressed hBMSCs. Moreover, using SB203580, a specific inhibitor of p38 MAPK, also activated the CB1-inhibited Erk1/2 signaling pathway in hBMSCs, but could not affect the CB1-enhanced JNK signaling pathway. These results suggested that the activation of p38 MAPK depended on the activation of JNK by CB1, which is a downstream molecule of the JNK signaling pathway, and Erk1/2 is a downstream molecule of the p38 MAPK signaling pathway after CB1 activation in hBMSCs. Next, after the addition of AM251, SB203580, and SP600125 in hBMSCs, the expressions of *Nrf1* and *Nrf2* were decreased, suggesting that CB1 may enhance mitochondrial biogenesis through p38 MAPK and JNK signaling pathways.

## Conclusion

Our findings suggested that CB1 increased the osteogenic differentiation potential of hBMSCs by targeting mitochondrial energy metabolism including OCR, MMP, and mitochondrial biogenesis under inflammatory conditions. Mechanismly, CB1 regulated mitochondrial energy metabolism of hBMSCs by activating of JNK signaling pathway and p38 MAPK signaling pathway, and further inhibiting the Erk1/2 signaling pathway. Thus, our study proved the potential role of CB1 in MSCs functional regulation and periodontal tissue regeneration, and provide a new target for bone tissue regeneration under inflammatory conditions.

## Supplementary Information

**Additional file 1: Table S1.** The primers for specific genes used in Real-time RT-PCR.

**Additional file 2: Fig. S1**. Depletion of CB1 by CB1 shRNA1 inhibited the expressions of osteogenic marker genes in BMSCs. Knockdown of CB1 by CB1 shRNA1 in BMSCs. Real-time RT-PCR results of the *RUNX2* (A), *ALP* (B), *OPN* (C), and *OSX* (D) expressions after knock-down of CB1 in BMSCs. GAPDH was used as an internal control. Error bars represent the SD (*n* = 3). *P ≤ 0.05; **P ≤ 0.01.

**Additional file 3: Fig. S2**. CB1 knockdown by CB1 shRNA2 inhibited the osteogenic differentiation of BMSCs. (A) Western blot results showed the knockdown efficiency of CB1 shRNA2 in BMSCs. β-actin was used as an internal control. (B) ALP activity assay. (C) Alizarin red staining. (D) OD values of the alizarin red staining. (E) Calcium quantitative analysis. Error bars represent the SD (*n* = 3). *P ≤ 0.05.

**Additional file 4: Fig. S3**. Over-expression of CB1 promoted the expressions of osteogenic marker genes in BMSCs. Real-time RT-PCR results of the *RUNX2* (A), *ALP* (B), *OPN* (C), and *OSX* (D) expressions after over-expression of CB1 in BMSCs. GAPDH was used as an internal control. Error bars represent the SD (*n* = 3). *P ≤ 0.05; **P ≤ 0.01.

## Data Availability

The data supporting the conclusions of this article are all online.

## Abbreviations

ALP: Alkaline phosphatase
CB1: Cannabinoid receptor I
CoQ10: Coenzyme Q10
DPSCs: Dental pulp stem cells
ECS: Endogenous cannabinoid system
ETC: Electron transport chain
GPCR: G protein-coupled receptor
HA: Haemagglutinin
hBMSCs: Human bone marrow mesenchymal stem cells
INF-γ: Interferon-gamma
MMP: Mitochondrial membrane potential
MSCs: Mesenchymal stem cells
mtDNA: Mitochondrial DNA
OCR: Oxygen consumption rate
OXPHOS: Oxidative phosphorylation
PDLSCs: Periodontal ligament stem cells
PKA: Protein kinase A
RANK: Nuclear factor-κB
ROT: Rotenone
RT-PCR: Reverse transcriptase-polymerase chain reaction
sAC: Soluble adenylyl cyclase
TNF-α: Tumor necrosis factor-alpha
