# Supplementary material for: The cannabinoid receptor I (CB1) enhanced the osteogenic differentiation of BMSCs by rescue impaired mitochondrial metabolism function under inflammatory condition
Source: Stem Cell Res Ther. 2022 Jan 21;13:22. doi: 10.1186/s13287-022-02702-9 (PMC8781353; doi:10.1186/s13287-022-02702-9)
Supplement: Supplementary file 1 — Additional file 1: Table S1. The primers for specific genes used in Real-time RT-PCR. [file 13287_2022_2702_MOESM1_ESM.doc]

**Supplementary table 1. The primers for specific genes used in Real-time RT-PCR**

| **Specific Genes** | **Target Sequences** |
| --- | --- |
| GAPDH-Forward  GAPDH-Reverse  CB1-Forward  CB1-Reverse  Nrf1-Forward  Nrf1-Reverse  Nrf2-Forward  Nrf2-Reverse  RUNX2-Forward  RUNX2-Reverse  OPN-Forward  OPN-Reverse  ALP-Forward  ALP-Reverse  OSX-Forward  OSX-Reverse | 5’‑CGGACCAATACGACCAAATCCG-3’ 5’‑AGCCACATCGCTCAGACACC-3’ 5’-CGGACCAATACGACCAAATCCG-3’ 5’-AGCCACATCGCTCAGACACC-3’  5’-AGGAACACGGAGTGACCCAA-3’  5’-TATGCTCGGTGTAAGTAGCCA-3’  5’-TCTCCATATCCCATTCCC-3’  5’-AAGGTGCTGAGTTGTTTT-3’  5’-TGGTTACTGTCATGGCGGGTA-3’  5’-TCTCAGATCGTTGAACCTTGCTA-3’  5’-CTCCATTGACTCGAACGACTC-3’  5’-CAGGTCTGCGAAACTTCTTAGAT-3’  5’-ACCACCACGAGAGTGAACCA-3’  5’-CGTTGTCTGAGTACCAGTCCC-3’  5’‑CCTCCTCAGCTCACCTTCTC-3’ 5’‑GTTGGGAGCCCAAATAGAAA-3’ |
