# Supplementary figures and images for: The cannabinoid receptor I (CB1) enhanced the osteogenic differentiation of BMSCs by rescue impaired mitochondrial metabolism function under inflammatory condition
Source: Stem Cell Res Ther. 2022 Jan 21;13:22. doi: 10.1186/s13287-022-02702-9 (PMC8781353; doi:10.1186/s13287-022-02702-9)

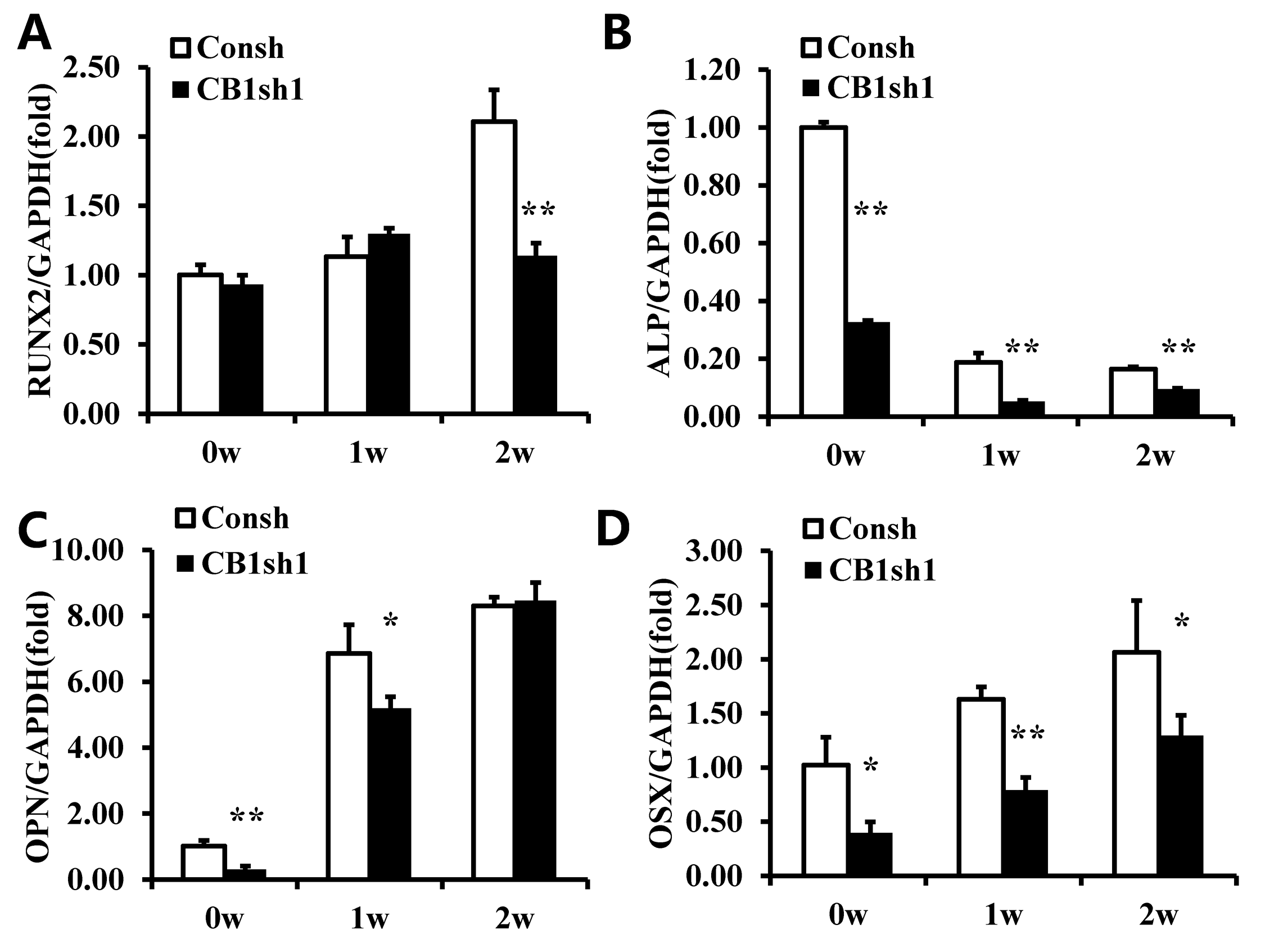

Supplement: Supplementary file 2 — Additional file 2: Fig. S1. Depletion of CB1 by CB1 shRNA1 inhibited the expressions of osteogenic marker genes in BMSCs. Knockdown of CB1 by CB1 shRNA1 in BMSCs. Real-time RT-PCR results of the RUNX2 (A), ALP (B), OPN (C), and OSX (D) expressions after knock-down of CB1 in BMSCs. GAPDH was used as an internal control. Error bars represent the SD (n = 3). *P ≤ 0.05; **P ≤ 0.01. [file 13287_2022_2702_MOESM2_ESM.tif]

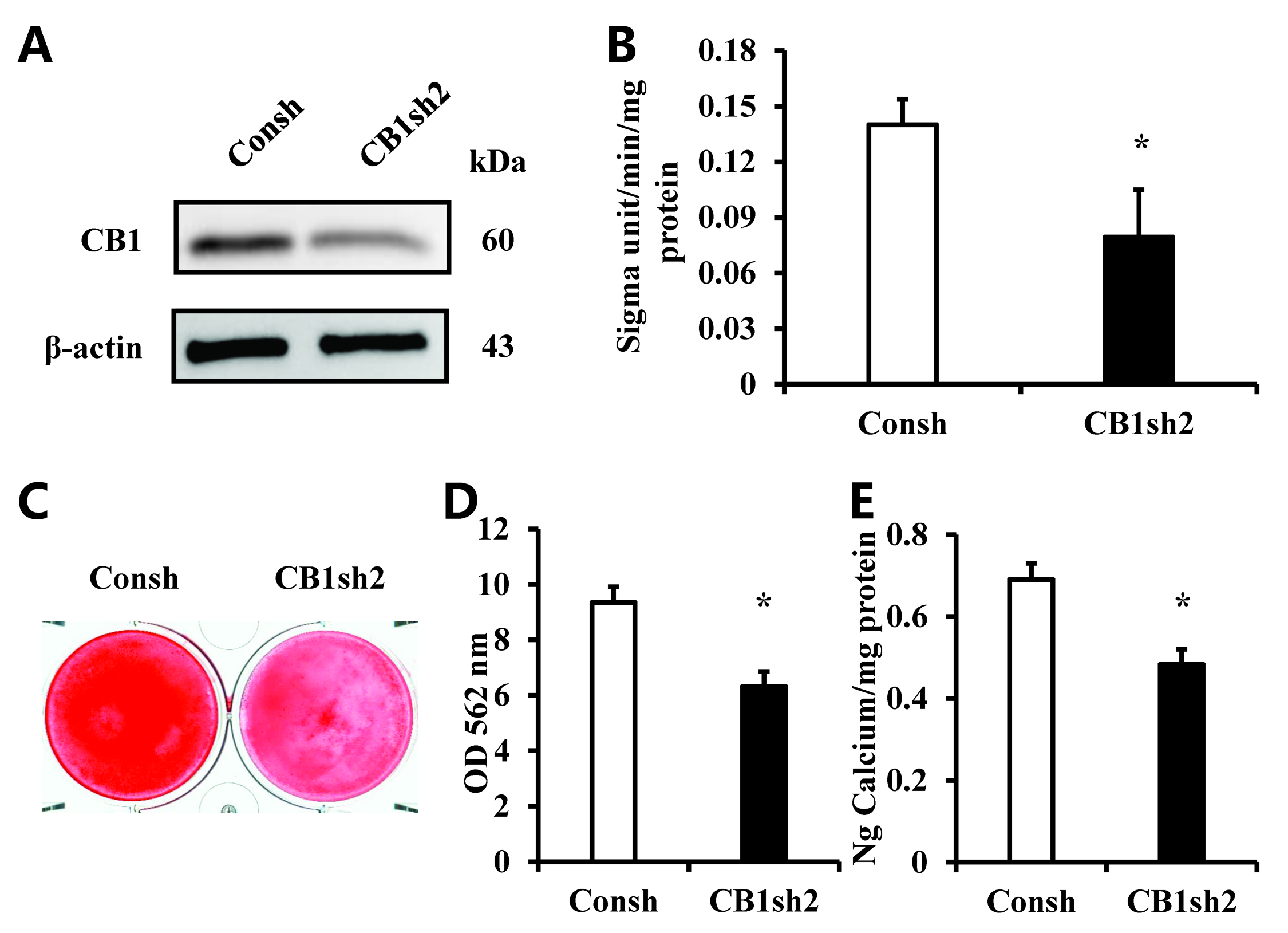

Supplement: Supplementary file 3 — Additional file 3: Fig. S2. CB1 knockdown by CB1 shRNA2 inhibited the osteogenic differentiation of BMSCs. (A) Western blot results showed the knockdown efficiency of CB1 shRNA2 in BMSCs. β-actin was used as an internal control. (B) ALP activity assay. (C) Alizarin red staining. (D) OD values of the alizarin red staining. (E) Calcium quantitative analysis. Error bars represent the SD (n = 3). *P ≤ 0.05. [file 13287_2022_2702_MOESM3_ESM.tif]

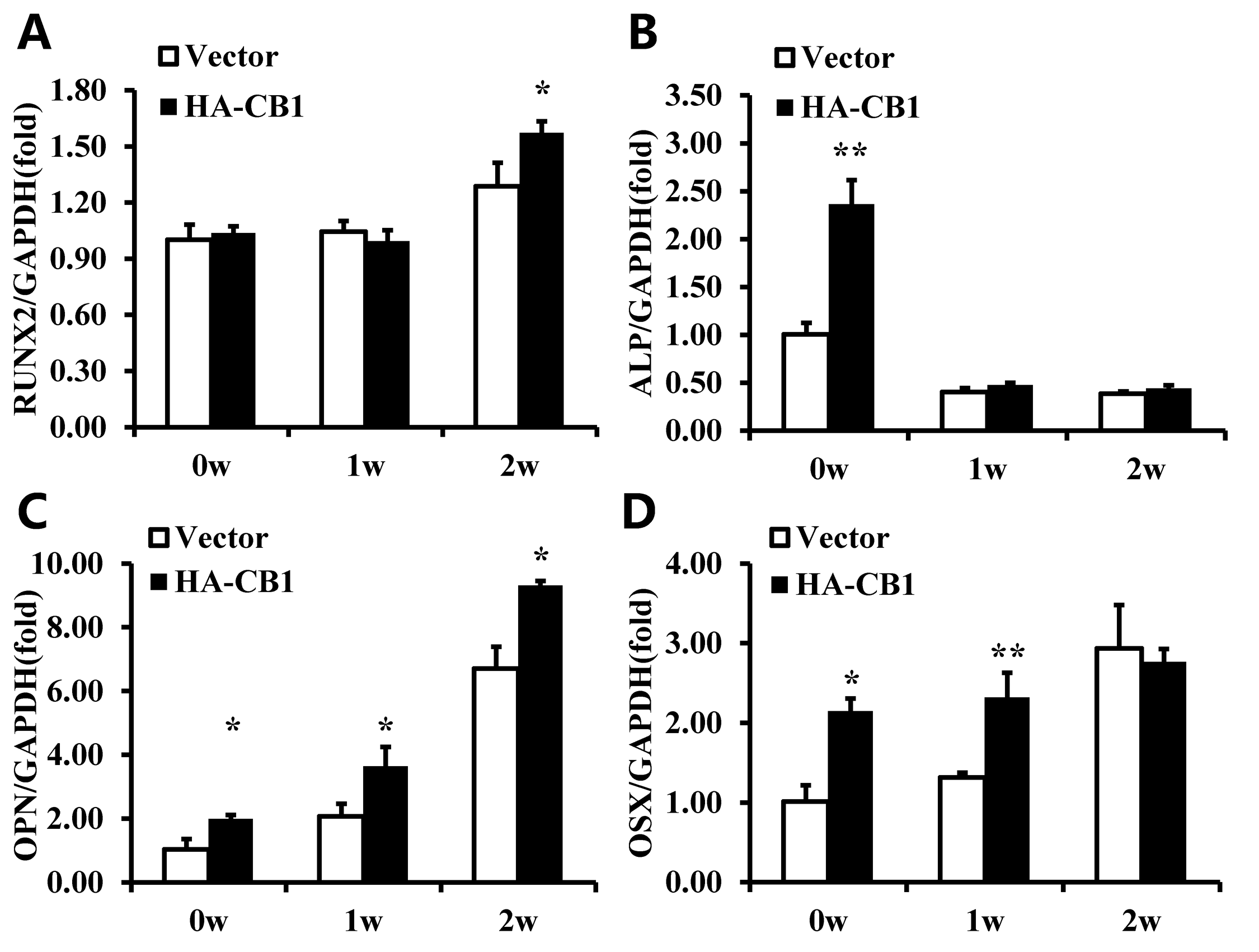

Supplement: Supplementary file 4 — Additional file 4: Fig. S3. Over-expression of CB1 promoted the expressions of osteogenic marker genes in BMSCs. Real-time RT-PCR results of the RUNX2 (A), ALP (B), OPN (C), and OSX (D) expressions after over-expression of CB1 in BMSCs. GAPDH was used as an internal control. Error bars represent the SD (n = 3). *P ≤ 0.05; **P ≤ 0.01. [file 13287_2022_2702_MOESM4_ESM.tif]
